# The Lamella Ostium Extent Mucosa (LOEM) system: a new classification and pilot study for endoscopic sinus surgery

**DOI:** 10.1007/s00405-024-09092-z

**Published:** 2024-12-02

**Authors:** Serafin Sanchez-Gomez, Daniel Martin-Jimenez, Ramon Moreno-Luna, Juan Maza-Solano, Christian Calvo-Henriquez, Alfonso del Cuvillo, Jose M. Villacampa-Auba, Alfonso Santamaria-Gadea, Ainhoa Garcia-Lliberos, Alvaro Sanchez-Barrueco, Gabriel Martinez-Capoccioni, David Lobo-Duro, Jaime Gonzalez-Garcia, Jose Palacios-Garcia, Rafael Fernandez-Liesa, Isam Alobid, Manuel Bernal-Sprekelsen

**Affiliations:** 1 Rhinology Unit, Department of Otolaryngology, Head and Neck Surgery, Virgen Macarena University Hospital, Dr Fedriani Av. 3, 41009 Seville, Spain; 2 Department of Otolaryngology, Head and Neck Surgery, Alava University Hospital, 01009 Alava, Spain; 3 Department of Otolaryngology, Hospital Complex of Santiago de Compostela, 15706 A Coruña, Spain; 4 Rhinology and Asthma Unit, Department of Otolaryngology, Jerez University Hospital, 11407 Jerez, Spain; 5 ENT and Cervicofacial Surgery Department, Fundacion Jimenez Diaz University Hospital, 28040 Madrid, Spain; 6 Rhinology Unit and Skull Base Unit, Department of Otolaryngology, Ramón y Cajal Hospital, Madrid, Spain; 7 Department of Otolaryngology, Valencia University General Hospital, 46014 Valencia, Spain; 8 Department of Otolaryngology, Hospital Universitario Marqués de Valdecilla, 39008 Santander, Spain; 9 Rhinology and Anterior Skull Base Unit, ENT Department, Hospital Universitario Miguel Servet, 50009 Saragossa, Spain; 10 Rhinology and Skull Base Unit, Department of Otorhinolaryngology, Hospital Clinic, 08036 Barcelona, Spain; 11 Department of Surgery, Seville University, Seville, Spain; 12 Continuing Education Master’s Program in Advanced Rhinology and Anterior Skull Base, International University of Andalucía, Seville, Spain; 13 President, Spanish Society of Otolaryngology and Head and Neck Surgery (SEORL-CCC), Madrid, Spain; 14 Past President, Spanish Society of Otolaryngology and Head and Neck Surgery (SEORL-CCC), Madrid, Spain; 15 Vicepresident, Spanish Society of Otolaryngology and Head and Neck Surgery (SEORL-CCC), Madrid, Spain; 16 President of the Rhinology Committee, Spanish Society of Otolaryngology and Head and Neck Surgery (SEORL-CCC), Madrid, Spain; 17 Board Member of the Rhinology Committee, Spanish Society of Otolaryngology and Head and Neck Surgery (SEORL-CCC), Madrid, Spain; 18 Marqués de Valdecilla Research Institute (IDIVAL) and University Hospital Marqués de Valdecilla, Santander, Spain

**Keywords:** Consensus, Nasal mucosa, Nasal surgical procedures, Paranasal sinuses, Rhinosinusitis

## Abstract

**Purpose:**

This study proposes the Lamella Ostium Extent Mucosa (LOEM) system as a compact and user-friendly classification for endoscopic sinus surgery (ESS), based on surgical bone extension and mucosal management, aiming to resolve inconsistencies in describing surgical techniques and extension levels, and to enhance comparability of outcomes in chronic rhinosinusitis (CRS).

**Methods:**

LOEM uses a lettering system representing a specific topographical level: L identifies the lamellae, O the ostia, E the opening of the sinus walls, and M the mucosal approach. Eleven CRS surgical cases were independently evaluated by seven rhinologists following a Delphi method in two consecutive rounds. Consensus was assessed using Cohen's kappa.

**Results:**

A substantial agreement was found among the experts (κ = 0.77) in the first round, although the M item only showed fair agreement (κ = 0.37). Clarifications for this item were given in the second round, after which, the overall agreement increased to κ = 0.81 and to κ = 0.79 for the M item. A decrease in agreement from substantial to moderate for O and E items in the second round was found. Test–retest analysis showed an almost perfect agreement (92.96%, κ = 0.82). In this study, a web-based app is provided to assist with the regular use of the LOEM system.

**Conclusions:**

The LOEM system provides a compact, comprehensive code for ESS, integrating anatomical and functional aspects to represent surgical techniques described so far. This system may be suitable for facilitating communication between surgeons and collecting robust labeled data, hopefully leading to further standardization and validation of surgical approaches in future CRS studies.

**Supplementary Information:**

The online version contains supplementary material available at 10.1007/s00405-024-09092-z.

## Introduction

For those who suffer from chronic rhinosinusitis (CRS) and fail to improve with appropriate medical treatment, endoscopic sinus surgery (ESS) remains a viable option [1]. Conventional functional ESS (FESS) addresses CRS by selectively removing obstructive osteomeatal structures and visible inflamed mucosa [2, 3]. FESS aims to restore the normal sinus function by reopening the sinus drainage pathways through limited or minimally invasive access, leading to suitable rates of disease control in non-severe patients [4, 5]. However, in CRS with nasal polyps (CRSwNP), severe mucosal inflammation, or comorbidities such as asthma or NSAID-exacerbated respiratory disease [6, 7], achieving positive surgical results can prove challenging.

In recent years, CRSwNP has been demonstrated to constitute a diffuse mucosal disease rather than a simple local inflammation, due to the high probability of revision, the long-term course of the disease, and the disturbed mucosal healing under type-2 inflammatory conditions [8]. As such, EPOS and ICAR’s most recent guidelines recognize the role of these novel surgical techniques, known as extended ESS (EESS), in specific conditions that require both a more extensive resection of bony sinuses and mucosal treatment [9, 10]. Types of EESS proposed in the literature are: nasalization, which targets polyp burden reduction and airflow improvement through extensive ethmoidectomy [11]; full-house FESS, which offers a comprehensive clearance of macroscopic mucosal disease and restoration of sinus function [12]; reboot surgery, which addresses persistent or recurrent CRS through a total removal of the mucosa of the nasal and paranasal sinuses [13]; and regenerative surgery (i.e., mucoplasty), which combines reboot surgery with mucosal regeneration techniques to promote local tissue healing [14, 15]. Interestingly, EESS has become especially relevant in severe and recalcitrant phenotypes in which classical FESS has been unsuccessful or when multiple revision surgeries have been required [16, 17].

However, despite such advances, and due to heterogeneous and divergent results published in the literature to date [18–20], there remains an ongoing debate surrounding the actual benefit of such extensive surgeries in the control of CRS. This variance could be partially explained by the fact that the different coexistent surgical techniques have no unequivocal definition, leading to different extents of osseous and mucosal resection being dependent on factors such as patient phenotype, the extent of the disease, and an individual surgeon’s preference [21]. Although some previous classification systems proposed for the surgery of the frontal sinus, such as Draf and Extent of Endoscopic Frontal Sinus Surgery (EFSS) classifications, have shown to provide useful criteria to compare results at this anatomical level [22, 23], the lack of unified consensus in CRS still poses several challenges and limitations, preventing the comparability and validation of surgical results in the field [24]. A comprehensive and detailed delineation of the surgical procedures as a whole, rather than independently for each paranasal sinus, would lead to a better comprehension of the clinical outcomes achieved.

In response to the challenges posed by existing classification systems, the Lamella Ostium Extent Mucosa (LOEM) system has been proposed for ESS [25]. This innovative classification is designed to address significant gaps in the interpretation of surgical outcomes in CRS. LOEM is based on a compact yet descriptive representation of the surgery performed on different nasal structures (i.e., lamellae, ostium, and sinus walls). By separating mucosal management from surgical extension, LOEM describes surgical procedures from both anatomical and functional perspectives, enhancing clarity and precision.

This work proposes and develops a preliminary validation of the LOEM system among experts in ESS classification. Its aim is to improve the standardization, precision and comparability of individual surgical procedures, which should enable the collection of more robust data, allow for more meaningful comparisons across studies and create effective communication among surgeons, ultimately improving patient care.

## Material and methods

**Fig. 1:**
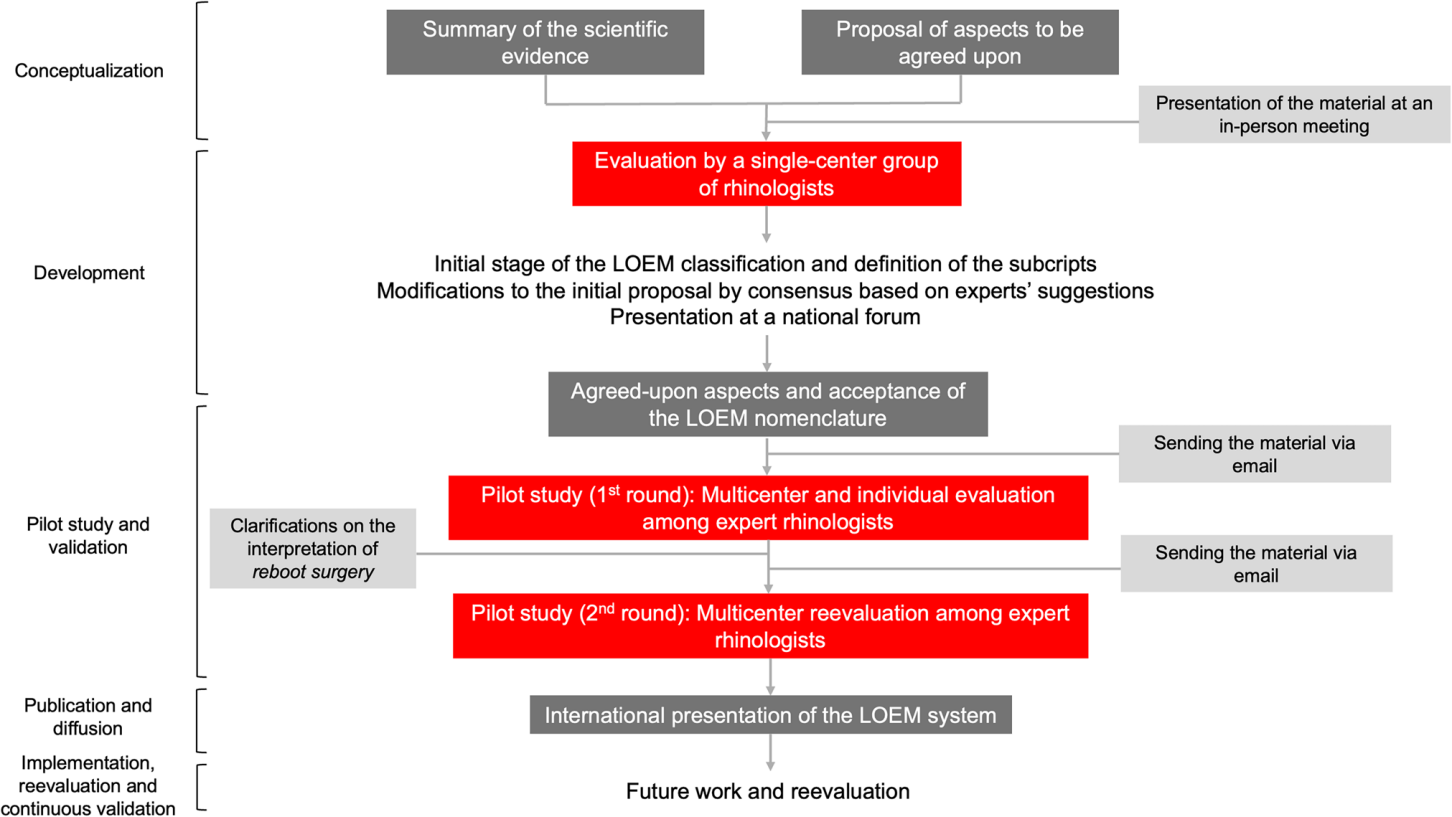
Flowchart of the LOEM classification development process

A classification system under the acronym LOEM is developed in this work. The systematic process followed for its development and validation is shown in detail in Fig. 1. This classification offers a standardized method for coding the extent of ESS performed and comprehensively describes the procedures conducted on the bony structures and mucosa of the sinonasal cavity. The LOEM classification is applied by the surgeon performing the procedure, with no need for post-surgical complementary tests.

### Rationale behind the LOEM classification system

The LOEM classification system aims to group similar surgical procedures into types based on two criteria: anatomy and functionality. The anatomical criterion considers the anteroposterior longitudinal extent of the surgery involving the lamella (L) and the enlargement/opening of ostia (O). The trend toward greater extension of the ESS also necessitates highlighting the removal of the sinus wall (E) in the anatomical criteria. The functionality criterion (M) is based on the intended outcome of the procedure: preserve sinus aeration and mucociliary function (limited and extended functional surgeries), completely target the mucosa (reboot surgery) or perform regenerative surgery (mucoplasty).

### The LOEM classification system

**Fig. 2:**
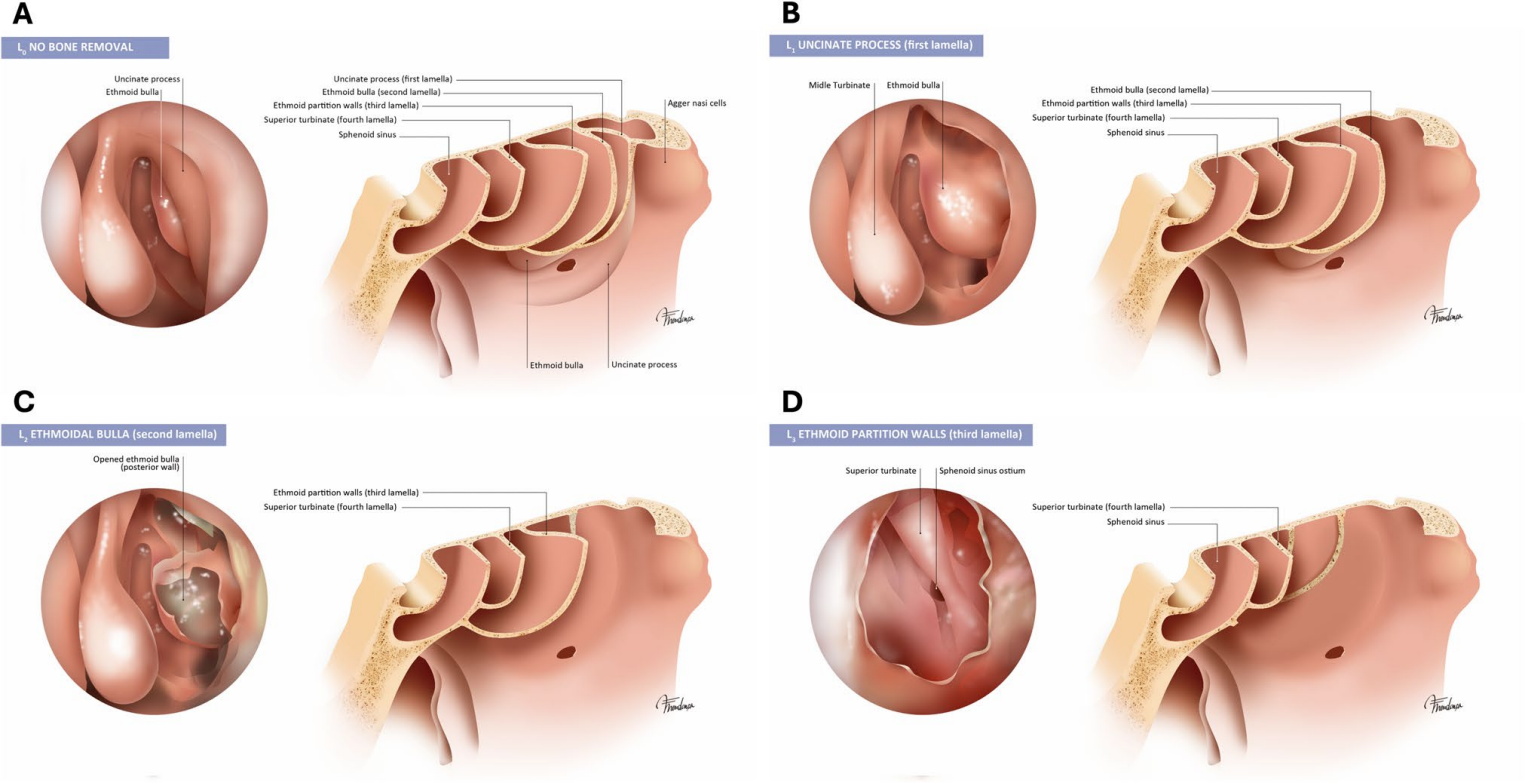
Longitudinal extent of surgery based on the number of removed lamellae. **A** L_0_: No intervention is performed on the anterior and posterior ethmoid structures. This may occur when no intentional intervention on bones is required (e.g., simple polypectomy). **B** L_1_: First lamella. This includes the removal of the uncinate process and the Agger nasi cell, along with other anterior ethmoidal cells that are superior to the Agger nasi, if present. **C** L_2_: Second lamella. This includes the removal of the ethmoid bulla, in addition to any suprabullar and retrobullar cells and recesses present. **D** L_3_: Third lamella. This includes the opening of the vertical plate of the basal lamella of the middle turbinate and the removal of any bony septa of the posterior ethmoidal cells [25]

**Fig. 3:**
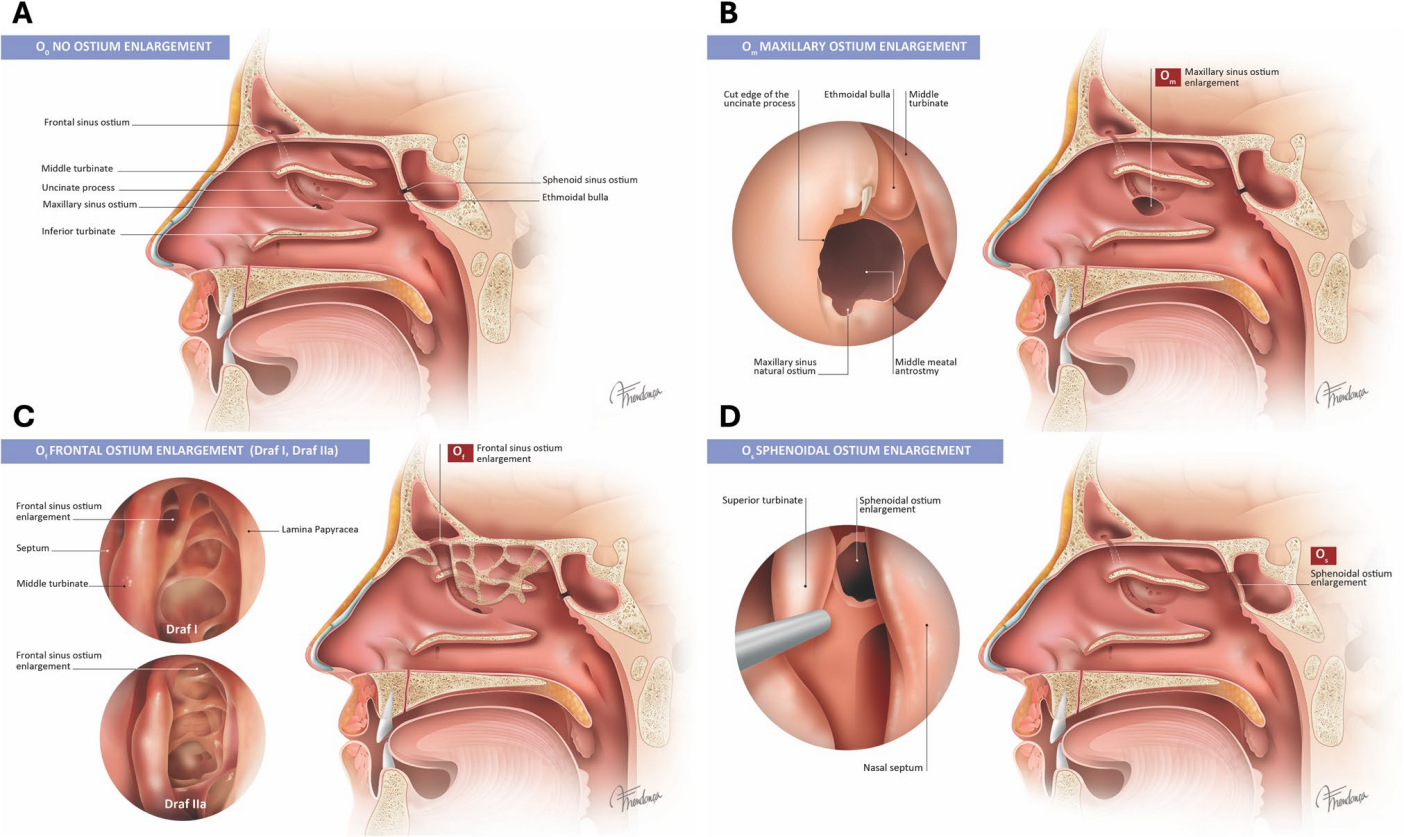
The second bony criterion of the LOEM system introduces the enlargement of natural sinus ostia or opening. **A** O_0_: No intervention is performed on any of the sinus ostia. This category includes cases that have a permeable ostium, or cases in which the ostium/opening was intentionally left untouched. **B** O_m_: Enlargement of the maxillary sinus ostium. **C** O_f_: Draf I and IIa frontal sinus surgery types or grades 0–4 of EFSS classification. **D** O_s_: Enlargement techniques performed on the sphenoid sinus ostium, regardless of the specific surgical approach used [25]

**Fig. 4:**
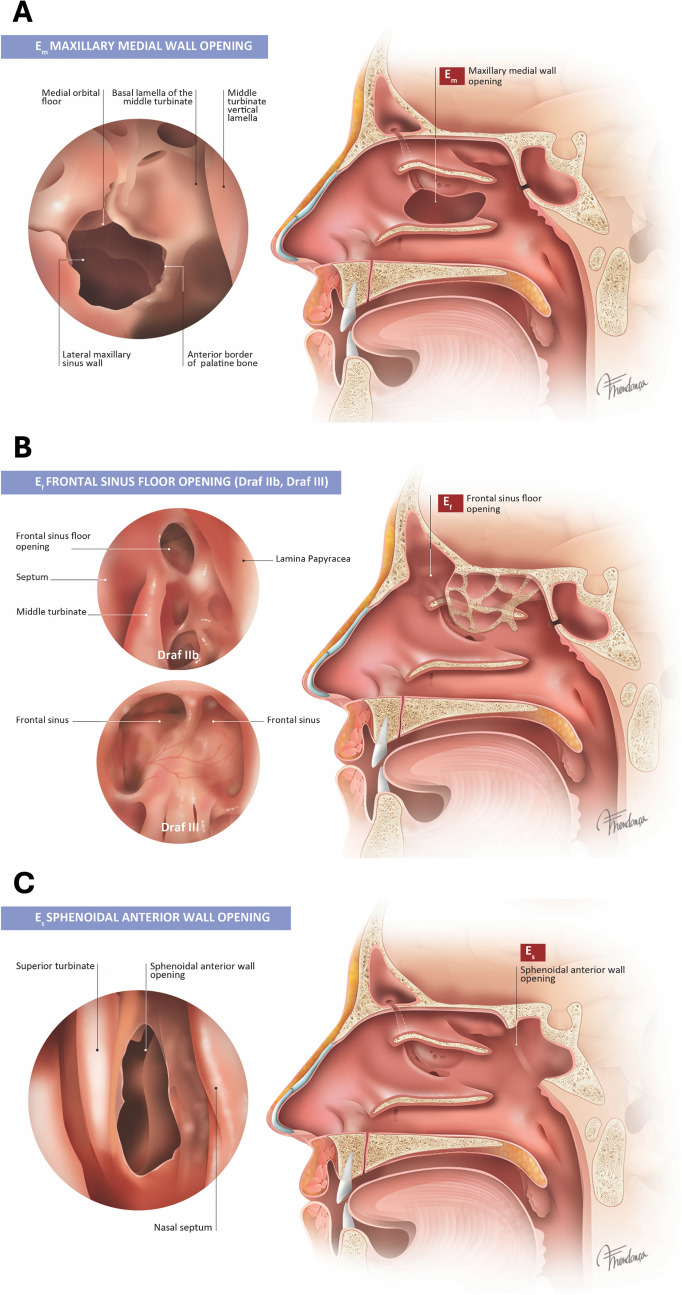
Enlargement of opening of walls beyond ostial/opening. **A** E_m_: Widening of the medial wall of the maxillary sinus beyond middle meatal antrostomy, including partial or total resection of the fontanelle. **B** E_f_: significant opening of the frontal sinus floor through Draf IIb or Draf III or grades 5–6 of EFSS classification. **C** E_s_: Enlargement of the anterior wall of the sphenoid sinus, including bone removal around the ostium [25]

In this way, LOEM uses a lettering system to differentiate specific topographic levels of surgical intervention, based on widely-accepted anatomical nomenclature [26]. A subscript code identifies the specific anatomical structures associated with each topographic level, as explained below. For the sake of clarity and visualization, Table 1 shows the letters and subscripts used in the LOEM classification system. Figures 2, 3, and 4 depict the technical procedures described in LOEM on each of the anatomical structures of the nose and paranasal sinuses.

**Table 1:** Construction of the LOEM classification system

Bone removal	Lamella removal	L_0_	No bone removal	No intervention is performed on the anterior and posterior ethmoid structures. This may occur when no intentional intervention on bones is required (e.g., simple polypectomy)
		L_1_	Uncinate process	The first lamella involves the removal of the uncinate process and the *agger nasi* cell, along with other anterior ethmoidal cells that are superior to the *agger nasi* if present
		L_2_	Ethmoidal bulla	The second lamella refers to the removal of the ethmoid bulla, as well as any suprabullar and retrobullar cells and recesses that may be present
		L_3_	Ethmoid partition walls	The third lamella includes the opening of the vertical plate of the basal lamella of the middle turbinate (MT) and the removal of any bony septa of the posterior ethmoidal cells
		L_xa_	Lamella absent	The subscript 'a' is added to denote the absence of a lamella removed during previous surgeries: L_xa_, where 'x' can take the values '1', '2', and '3', representing the missing lamella. For example, the notation L_1a,2a,3_, indicates a patient who has undergone prior ESS in which the first and second lamellae were removed, and for whom the third lamella will be removed in the current surgery
	Ostium enlargement	O_0_	No ostium enlargement	No intervention is performed on any of the sinus ostia. This category includes cases that have a permeable ostium, or cases in which the ostium/opening was intentionally left untouched
		O_m_	Maxillary ostium enlargement	Enlargement of the maxillary sinus ostium
		O_f_	Frontal ostium enlargement (Draf I, Draf IIa)	Draf I and IIa frontal sinus surgery or Grades 0 to 4 of the Classification of the Extent of Endoscopic Frontal Sinus Surgery (EFSS) [22, 23]
		O_s_	Sphenoidal ostium enlargement	Enlargement techniques performed on the sphenoid sinus ostium, regardless of the specific surgical approach used (modified type I sinusotomy, not extending upward to the skull base [36])
		O_xa_	Ostium previously enlarged	The subscript 'a' is added to indicate a previously enlarged ostium/opening. The ‘x’ in the subscript should be replaced by the enlarged ostium
	Extension approach	E_0_	No extended approach	No extended intervention is performed on the walls of any sinus
		E_m_	Maxillary medial wall opening	Widening of the medial wall of the maxillary sinus beyond middle meatal antrostomy, including partial or total resection of the fontanelle (transnasal endoscopic partial maxillectomy type 1 [35])
		E_f_	Frontal sinus floor opening (Draf IIb, Draf III)	Significant opening of the frontal sinus floor through Draf IIb or Draf III or grades 5–6 of EFSS classification [22, 23]
		E_s_	Sphenoid anterior wall opening	Enlargement of the anterior wall of the sphenoid sinus, including bone removal around the ostium (type II and III sinusotomies [36])
		E_t_	Turbinate resection	Partial removal, trimming or ablation of the MT
		E_xa_	Sinus wall absent	The subscript 'a' is added to indicate a previously removed sinus wall. The ‘x’ in the subscript should be replaced by the enlarged sinus wall
Mucosal removal		M_0_	No mucosal removal	Cases with no mucosal removal, except for the enlargement of the sinus ostia/openings
		M_f_	Functional. Only diseased mucosa removal. Preservation of mucociliary function and sinus aeration	The main objective is to preserve mucociliary function and ventilation of the paranasal sinuses, regardless of the performance on the bone. It also aims to facilitate the installation of topical treatments. This category encompasses the original criterion of functional ESS aimed at removing only macroscopically irreversibly diseased mucosa
		M_r_	Reboot approach. Complete mucosal removal	The objective is to replace all sinus mucosa with healthy local mucosa, regardless of the intervention on the bone. This procedure is considered a reboot approach following a complete mucosal removal
		M_m_	Mucoplasty + reboot approach (regenerative surgery)	The objective is to create neomucosa from freely grafted healthy mucosa covering a significant part of the excised mucosal area in addition to radical ESS. This type of surgery is classified as mucoplasty regenerative surgery

Some of the information contained in this table has already been previously published [25]

#### Lamella

The bone structures attached to the lateral wall of the nasal capsule are known as lamellae. In the proposed system, the letter L and the numerical subscripts are used to identify the lamella removed during surgery (see Table 1). In addition to the aforementioned numerical subscripts, the subscript 'a' can also be added to denote the absence of a lamella removed during previous surgeries: L_xa_, where 'x' can take the values '1', '2', and '3', representing the missing lamella. For example, the notation L_1a,2a,3_, indicates a patient who has undergone prior ESS in which the first and second lamellae were removed, and for whom the third lamella will be removed in the current surgery.

#### Ostium

The letter O is used to describe openings or enlargements of up to a diameter of 1 cm in the natural ostia, a common surgical procedure used to improve sinus drainage and aeration. The subscripts (shown in Table 1) indicate the sinus involved. Additionally, the subscript 'a' can also be attached to the aforementioned subscripts to indicate a previously enlarged ostium opening, i.e. O_xa_, where 'x' can take the values 'm', 'f', and 's', depending on the sinus involved.

#### Extent

The letter E is used to describe enlargements of natural sinus ostia with a diameter greater than 1 cm. Similarly, the subscripts indicate the sinus or turbinate involved (Table 1). The subscript 'a' can be attached to the aforementioned subscripts, i.e., E_xa_ to indicate that some of the abovementioned sinus wall or middle turbinate has been removed in previous surgeries. Specifically, more extensive transnasal endoscopic partial maxillectomies (types 2–4) are recorded under the subscripts E_m_ and should be specified as a supplementary approach in the observations.

#### Mucosa

Finally, the letter M is related to mucosal function and can be further divided into four categories, depending on the extent of mucosal resection, as depicted in Table 1.

The LOEM system permits unilateral or bilateral ESS descriptions and suggests utilizing P-ESS for primary surgeries and R-ESS for revision surgeries. In cases of R-ESS, the absence of previously performed structures or procedures will be reported under the subscript 'a' (i.e., L_xa_, O_xa_, E_xa_). Previous approaches on the lamella (L), ostium (O) and extension (E) will be considered part of the current surgical process, allowing grading types 2, 3 or 4 solely by the approach taken on the mucosa (M).

### Pilot study

To assess the feasibility of the LOEM system to describe routine surgeries, a pilot study was conducted by seven different expert rhinologists. Four of whom are members of the Spanish Commission of Rhinology, Allergy and Anterior Skull Base Surgery, two are members of the expert group of the European Rhinologic Society that developed the EPOS guidelines and the remaining expert is on the Advisory Board of the European Rhinologic Society. All seven rhinologists had more than 10 years of experience performing ESS in CRS patients (> 60 cases per year). The experts were instructed to analyze a total of eleven videos of a unilateral nasal fossa ESS. Written consent was secured, identifying data was anonymized and access was limited to essential researchers to protect the experts' confidentiality. The experts were blinded to the characteristics of the patients, the surgeon who performed the surgery, and the responses provided by the other raters. Two rounds of different case surgeries were evaluated by means of a Delphi method, based on an initial document with a detailed description of the LOEM classification system. Five cases were assessed in the first round and six cases in the second round. The selected cases were primary surgeries (thus items L_xa_, O_xa_ and E_xa_ were not evaluated) and represent a wide range of CRS phenotypes, allowing for greater generalizability of the findings. Additionally, a geographic diversity was obtained by selecting cases from different surgical centers, and the complexity of the cases was balanced to include both basic and expanded surgeries. The picked surgical videos had not been previously edited, were accessible to all experts involved in the Delphi method and had a video resolution of 720p (HD) or higher. Cases involving endoscopic polypectomy or balloon sinuplasties (i.e., surgeries that do not modify bony structures) were not analyzed, hence items O_0_ and E_0_ were not collected. For each surgical case, 15 items based on the LOEM system were defined in each round. After the first round, the evaluations and comments received were shared among the experts through a detailed report. Relevant clarifications regarding the mucosal resection of reboot surgery technique as initially described in [13, 27] were included in this report, which was shared among the experts in the second round.

Additionally, to evaluate the consistency and reliability of the results, a test–retest analysis was conducted with the same rhinologists at two different times. Six months after the initial evaluation, the experts were asked to review the second-round videos again. This interval ensured the stability of the constructs evaluated by the LOEM classification, minimized potential bias arising from learning or familiarity with the classification, and guaranteed that the observed differences in scores reflected real variations in decisions rather than the effects of repeated evaluations over a short period. This analysis measured how well the two sets of evaluations matched, while also accounting for the agreement that could occur by chance.

### Statistical analysis

The number of cases required for the pilot study was determined through a statistical power analysis for z tests, based on an expected alternative hypothesis of k ≥ 0.7, a desired significance level (α) of 0.05 and a power (1– β) of 0.80. For this calculation, we used the G*Power software (v 3.1.9 for Macintosh). Given the large number of LOEM items to be completed for each surgical case, a group of observers larger than estimated was used, with the aim of improving the measurement of variability.

Each variable of the LOEM system was codified as a dichotomous variable (yes or no). The consensus on LOEM values and the test–retest analysis provided by the experts for the different cases was analyzed using Cohen’s kappa coefficient κ, which ranges between 0 and 1. This test provided a statistically sound measure of how well the LOEM classification can be consistently used across different surgeons. Cohen’s recommendations for kappa values were used: values ≤ 0 indicate no agreement, 0.01–0.20 none to slight, 0.21–0.40 fair, 0.41–0.60 moderate, 0.61–0.80 substantial, and 0.81–1.00 almost perfect agreement. Limitations in the LOEM classification system were identified for responses with fair agreement (i.e., κ < 0.4) [28]. Confidence intervals for Kappa index were calculated using the Reichenheim approximation [29]. Data accuracy was ensured through expert reviews (intra- and inter-rater), Kappa coefficient analysis, and cross-checks to align the data with the LOEM classification criteria. Internal audits further reinforced reliability. A *p* value of 0.05 was considered significant in our analysis. Data was analyzed with Stata (StataCorp. 2023. Stata Statistical Software: Release 18. College Station, TX: StataCorp LLC).

## Results

### ESS techniques published to date

Table 2 provides a detailed description of the most representative ESS techniques reported so far. The surgical features are delineated based on the anatomical structures within the sinonasal cavity, with careful distinction made between procedures performed on bony structures and mucosa. For these surgeries, a description based on the LOEM system is proposed (Table 2).

**Table 2:** Main types of ESS approaches published to date thorough description of their characteristics and anatomical structures involved

	Functional endoscopic endonasal surgery (FEES)	Nasalization (radical ethmoidectomy)	Full-house FESS (FHFESS)	Reboot surgery	Regenerative surgery (reboot surgery plus mucoplasty)
Rationale	Conservative approach targeting osteomeatal complex disease (OMC)	Complete removal of the vestigial non-olfactory ethmoid mucosa based on the evo-devo theory	To treat affected sinuses (CT images), regardless of symptoms, complete removal of ethmoidal lamellae prevents obstruction and supports postoperative diagnostics and treatment	To remove all sinus mucosa, microbiota, and immune dysfunction, and promote healthy re-epithelialization from preserved nasal mucosa, starting with the inferior and MTs and septum	To encourage healthy epithelial regrowth and reduce polyp risk after mucosa removal, tissue from a non-polypoid donor site is used to aid re-epithelialization
Objective	To clear diseased ethmoid clefts, restore sinus ventilation and drainage, while minimizing tissue damage	Marsupialization of the ethmoid, maxillary, sphenoid, and frontal sinuses into the nasal cavities	FHFESS is a term used to describe uncinectomy, maxillary antrostomy, total ethmoidectomy, wide sphenoidotomy, and a Draf IIa frontal sinusotomy	To completely remove all mucosa from all sinuses (ethmoid, maxillary, sphenoid, frontal), while leaving the periosteum where possible	To promote the regeneration of tissue and facilitate re-growth of epithelial cells, regenerative surgery can be performed using a graft
Mucosa	Disease is targeted for removal in key areas of the anterior ethmoid and middle meatus. Preservation of as much mucosa as possible	Complete removal of the ethmoid mucosa, preserving mucosa in the large sinuses and around the frontal ostia. Mucosal release ensures precise navigation	Targeted removal of disease from key areas of the ethmoid and middle meatus	Complete removal	Complete removal using a mucosa release technique
Uncinectomy	Performed systematically	Performed systematically	Performed systematically	Performed systematically	Performed systematically
Ethmoidal bulla	Once the cell walls are fractured, they are removed	Once the cell walls are fractured, they are removed	Once the cell walls are fractured, they are removed	Once the cell walls are fractured, they are removed	Circumferential dissection and complete removal
Middle turbinate (MT)	Preservation is preferred	Systematically removed. Spared whenever possible but to preserve exclusively the mucosa covering its medial side within the olfactory cleft, after updating nasalization	Consider the possibility of medializing the MT or securing it to the septum through the induction of synechiae	Preserved as much as possible as a landmark, except for the areas that are damaged by the disease or the anterior portions that require removal during the Draf III procedure	Preservation preferred
Vertical plate of the MT	The basal lamella is perforated to enter the posterior ethmoid cells whenever needed	The basal lamella is opened to enter the posterior ethmoid cells and the opening is enlarged	The basal lamella is perforated to enter the posterior ethmoid cells and the opening is enlarged	The basal lamella is perforated to enter the posterior ethmoid cells and the opening is enlarged	The basal lamella is perforated to enter the posterior ethmoid cells and the opening is enlarged
Ethmoid bony lamellae	On demand	Removed systematically	Removed systematically	Removed systematically	Removed systematically
Middle meatal antrostomy	On demand	Large opening of the maxillary ostia, leaving mucosa intact	As large as possible	Wide antrostomy	Limited enlargement
Maxillary sinus mucosa	Localized irreversible disease is removed to the periosteum. Frequently, apparently irreversible mucosal disease resolves	Conservation of maxillary sinus membrane and ostium whenever possible in nasalization updated	On demand	Complete clearance	Complete clearance
Ethmoid sinus mucosa	On demand	Completely removed systematically	Removed systematically to the periosteum	Complete clearance, including the lamina orbitalis, skull base, and the lateral aspects of the MT	Complete clearance, including the lamina orbitalis, skull base, and the lateral aspects of the MT
Sphenoidotomy	On demand	Spared when possible	Wide	Wide	Wide
Sphenoid sinus mucosa	Preserved	Ostial conservation depending on the contents found	Preserved unless grossly abnormal	Should try to remove the diseased mucosa from the floor and medial parts of the sphenoid	Should try to remove the diseased mucosa from the floor and medial parts of the sphenoid
Frontal sinus opening	Clearing the frontal recess typically heals the sinus without requiring additional ostium enlargement	The circumferential mucosa of the ostium is to be conserved	Draf IIa, Draf III when indicated	Bilateral Draf IIa or III procedure with wide exposure of the posterior wall of the frontal sinus	Draf III
Frontal sinus mucosa	Preserved	Preserved	Frontal pathway clearance	Complete removal to the periosteum	Removed as possible
Adjunct procedures			Canine fossa trephination and frontal minitrephination		Mucoplasty from nasal floor
LOEM description	Conventional: L_1,2_O_m_M_f_ Extended: L_1,2,3_O_m,f,s_M_f_	L_1,2,3_O_0_E_0_M_r_	L_1,2,3_O_m,f,s_E_m,f,s_M_f_	L_1,2,3_O_m,f,s_E_m,f,s_M_r_	L_1,2,3_O_m,f,s_E_m,f,s_M_m_

The codification of these surgeries using the LOEM system is also shown. Some of the information contained in this table has already been previously published [24, 25]

### ESS types according to the LOEM classification

Following the LOEM classification system, our group proposes four types of surgery based on both anatomical and functional criteria. Table 3 groups several EES executions into four synthetic types that allows for a more simplified management of surgical approaches, especially useful when making comparisons across different studies and centers. Type 1, functional limited surgery, aims at restoring normal function while preserving anatomy. This involves minimal intervention to the lamellae and ostium, as well as limited mucosal removal to preserve mucociliary function and sinus aeration. Type 2, extended functional surgery, involves a more extensive intervention, including any approach to the lamellae and enlargement of the ostia and the sinus walls. However, since the main purpose remains to be the preservation of mucosal function, only the damaged mucosa is resected. Type 3, radical extended surgery, adopts a more extensive approach consisting of a complete resection of the mucosa (reboot) to prevent recurrence of polyps. Type 4, radical regenerative surgery, involves complete removal of the mucosa (reboot) followed by a mucoplasty to promote tissue regeneration.

**Table 3:** Categorization of ESS surgeries following LOEM classification

Type 1. Functional limited: at least 1 lamella (L) or 1 ostium (O) + no resection or 1 functional mucosal (M_f_)	
Any limited approach	L_0_, L_1_, L_2_, L_3_
Any ostium enlargement	O_0_, O_m_, O_f_, O_s_
Functional mucosal removal	M_0_, M_f_
Type 2. Functional extended: at least 2 lamellae (L) + at least 2 ostia (O) or 1 extended (E) + 1 functional mucosal (M_f_)	
Any lamellae approach	L_xa_, L_0_, L_1_, L_2_, L_3_
Any ostium enlargement	O_xa_, O_0_, O_m_, O_f_, O_s_
Any extended approach	E_xa_, E_0_, E_m_, E_f_, E_s_, E_t_
Functional mucosal removal	M_f_
Type 3. Radical extended: 3 lamellae (L) + 3 ostia (O) + at least 2 extended (E) + 1 radical mucosal (M_r_)	
Complete lamellae approach	L_xa_, L_0_, L_1_, L_2_, L_3_
Complete ostium enlargement	O_xa_, O_0_, O_m_, O_f_, O_s_
Any extended approach	E_xa_, E_0_, E_m_, E_f_, E_s_, E_t_
Reboot approach (complete mucosal removal)	M_r_
Type 4. Radical regenerative: 3 lamellae (L) + 3 ostia (O) + at least 2 extended (E) + 1 regenerative mucosal (M_m_)	
Complete lamellae approach	L_xa_, L_0_, L_1_, L_2_, L_3_
Complete ostium enlargement	O_xa_, O_0_, O_m_, O_f_, O_s_
Any extended approach	E_xa_, E_0_, E_m_, E_f_, E_s_, E_t_
Regenerative surgery (reboot + mucoplasty)	M_m_

Some of the information contained in this table has already been previously published [25]

In cases of revision surgeries (R-ESS), the absence of previously performed structures or procedures will be reported under the subscript 'a' (i.e., L_xa_, O_xa_, E_xa_). Previous approaches on the lamella (L), ostium (O) and extension (E) will be considered part of the current surgical process, allowing grading types 2, 3 or 4 solely by the approach taken on the mucosa (M)

### Web-based app design

Additionally, a web-based app has been developed to facilitate the description of the surgery using the LOEM system, which will be made publicly available to support further research and validation of the LOEM system upon publication of this manuscript. To assess the accuracy and usability of the web-based app, the preferred authors conducted a preliminary test to compare the types of surgery assigned by each case, leading to a qualitative analysis of outcomes and allowing for improvements and adjustments to be made to the final version. To use this web application, please access it through the following link: https://loem.netlify.app/

### Pilot study

#### Inter-rater agreement

Eleven surgical cases were evaluated by the experts in two different rounds: five cases in the first and six in the second. For each surgical case, the experts evaluated 15 different items of LOEM. Consequently, a total of 1.155 responses were given (Supplementary Table S1). The qualitative feedback from the experts after using the LOEM classification was also noted (Supplementary Table S2). The kappa index for the whole items and sub-categories L, O, E, and M are listed in Supplementary Table S3. The first round showed a substantial level of agreement on the overall items with κ = 0.77, with the lamella (L) showing a perfect agreement (κ = 1.0). Items O (κ = 0.66) and E (κ = 0.68) showed substantial agreement. The mucosal item, M, however, only showed fair agreement (κ = 0.37).

Between the rounds of the pilot study, various aspects were clarified for the evaluators. On one hand, the distinction between the enlargement of the ostium and the resection of the sinus wall was specified. It was clarified that the approaches to each sinus, through their natural drainage pathways, would include modifications to the ostium (item O) and, if the enlargement exceeded 1 cm, also to the wall (item E). To facilitate interpretation, the researchers also provided bibliographic references [22, 23, 35, 36]. Additionally, to enhance the understanding of the functionality criterion (item M) and to avoid discrepancies in the interpretation of the reboot surgery, its limitations in resection of the entire sinonasal cavity mucosa and the initial publication of the technique were referenced [13]. After clarifications, the second round showed an almost perfect agreement for overall items (κ = 0.81) and substantial agreement for M (κ = 0.79). This demonstrated great concordance for the LOEM system, and specifically for the management performed on the sinonasal cavity mucosa. In contrast, the agreement decreased from substantial to moderate for O and E items in this round, with κ = 0.52 and κ = 0.41, respectively.

#### Test–retest reliability

Additionally, test–retest results are summarized in Supplementary Table S4. The analysis showed varying levels of agreement between the same rhinologists at two different times. Overall, the agreement was 92.96%, with a Kappa value of 0.82, indicating strong consistency (p < 0.001). Specific items showed perfect agreement for L (100%, Kappa = 1.00, p < 0.001) and almost perfect agreement for M (95.24%, Kappa = 0.87, p < 0.001). Item E had a moderate agreement of 81.55% with a Kappa of 0.53 (p < 0.001), while item O showed no agreement (91.27%, Kappa = -0.05, p = 0.695) suggesting no reliability beyond chance.

### Challenges in LOEM system development

The definition of the O and E subscripts questioned the need to distinguish between an approach to the sinus ostium and one to the paranasal sinus wall. To resolve this, the researchers emphasized the importance of defining both concepts, as they are crucial for differentiating the various extents of ESS. Moreover, the initial classification of surgery types (types 1 to 4) posed a challenge. It was necessary to repeatedly adjust the alignment between bony extension and mucosal resection to achieve clinically coherent classifications. Additionally, in the final version of the LOEM system, some ESS variants are not included in the four defined types, though the authors believe these alternatives do not represent viable surgical approaches for treating CRS.

To further improve the LOEM classification for ESS in CRS, future multicenter validation studies, subgroup analyses and periodic updates based on new evidence are planned. Standardizing data collection, incorporating postoperative outcomes and comparing LOEM with other systems, along with the use of technologies such as artificial intelligence (AI), could further enhance its accuracy and efficacy.

## Discussion

This study aims to introduce the LOEM system as a compact but descriptive tool to standardize the extent of ESS performed in individual CRS cases, by providing descriptions of both the extent of the surgical approach and the mucosal treatment. With an evaluation of an expert, the LOEM system could be connected to diagnostic cases and surgical interventions allowing for a proof-of-concept in the field of Rhinology. Furthermore, the LOEM classification condenses expert surgical knowledge in CRS into a structured and simplified format, which may pave the way towards the collection of more comprehensive datasets in CRS.

### Need for CRS surgical standardization

There is an outstanding need for standardization of surgeries in CRS management [30]. Although various metrics are used to quantify radiologic [31], nasal polyp size [32] and endoscopic outcomes [33], few attempts have been made to comprehensively classify ESS. Some approaches exist in the literature for frontal sinus surgery [22, 23, 34], maxillary ostium enlargement [35] and sphenoid sinus ostium [36]; however, developing a more comprehensive system able to group interventions conducted in all sinuses remains a challenge. In 2013, the Japanese Rhinology Society proposed a classification system for ESS, which comprises five types: removal of the ostiomeatal complex (Type I), single sinus procedures (Type II), multiple sinus procedures (Type III), full-house surgeries (Type IV), and extended procedures beyond the sinus wall (Type V) [37]. Nevertheless, this system only numbers the procedure types without specifying the anatomical structures involved and does not describe relevant surgical details, such as the intervened paranasal sinuses, the extension of the ostium drainage, or the mucosa treatment. Another approach is the ACCESS system [38], which based on the Lund-Mackay score, provides a quantitative measure of the extent of ESS based on postoperative CT images. This classification allows the assessment of functional sinus patency and topical medication but does not define the actions performed on the mucosa. The LOEM system provides a comprehensive framework that encompasses the anatomical extent of surgery, ostium modifications and mucosal treatment. This enhances precision, evaluation and international comparability in endoscopic sinus surgery, while supporting the development of evidence-based guidelines for these procedures.

Although there is a consensus on the need for collecting more comprehensive datasets to analyze the true impact of different ESS, interchangeable definitions of complete, extended, and radical FESS based on non-standardized terminology persist in the literature, which hinder our ability to establish guidelines for patient management and evaluation of surgical outcomes [17, 25]. This ambiguity has led to misunderstandings about the extent of ESS and the management of the sinonasal mucosa. This is especially problematic at a time when new pathways for sinonasal mucosal inflammation are being identified, promoting more extended surgical techniques both on bony structures and the mucosa [21, 24]. In this sense, reboot surgery focuses on the complete removal of the mucosa under severe inflammatory states by promoting regrowth of healthy neomucosa [13]. In contrast with the classical functional mechanistic viewpoint, such a disruptive proposal has promoted the development of new complementary techniques such as regenerative surgery (i.e., mucoplasty), which promotes the regeneration of healthy mucosa from large free grafts from the floor of the nasal fossa [14, 15].

Similarly, CRS guidelines do not resolve the ambiguity of the terms functional and extended surgeries [9, 10, 39]. Some researchers consider that the objective of FESS should be the improvement of nasal breathing functionality [4], while others argue that it should encompass extensive procedures to facilitate effective delivery of intranasal medical therapy [3]. Additionally, there is a school of thought that limits functional surgery to the ethmoid and osteomeatal complex [2]. However, these guidelines fail to provide a clear delineation of the modifications carried out within FESS on nasal structures. The same ambiguity exists for the term extended surgery, also referred to as complete or radical ESS. Some authors, in an attempt to optimize surgical outcomes, use the term expanded FESS [20, 40]. Thus, specific descriptions of extended surgery procedures are provided by certain groups [12, 13, 18]. Conversely, other authors classify all previous definitions of extended surgery under the term conventional FESS, reserving extended or radical surgeries for procedures involving extensive sinus openings (such as frontal sinusotomy type DRAF III, mega-antrostomy or medial maxillectomy, or complete sphenoidotomy) [21]. Further consensus is needed to provide standardization of surgical procedures in CRS management.

### The LOEM classification system: properties

The LOEM coding system standardizes the surgical procedures not only based on the removal of anatomical structures such as the lamella L and ostium O, but also on the mucosa treatment (the letter E indicates whether the procedure was limited or extensive, and M indicates the procedure directed towards the sinonasal mucosa), which may prove useful for categorizing different types of ESS currently available in the clinical arena (Table 2). The LOEM classification lies on well-known anatomical structures and makes use of a simple descriptive lettering and numbering system. This proposed system is not influenced by surgical indication, preoperative or postoperative radiological evaluation or clinical outcomes, making it solely and exclusively limited to the definition of surgical procedures.

Accordingly, based on the extent of bone modifications and mucosal treatment used, LOEM proposes a description of surgical procedures though four compact types (Table 3). The LOEM system aims to overcome current heterogeneity by proposing a simple lettering system that simplifies the descriptions of various approaches reported in the literature and listed in Table 2. This classification attempts to address the lack of a validated tool to assess the extent of ESS and to facilitate research in this field [10, 39]. Moreover, this coding system is also useful for describing primary and revision surgeries on all sinuses and nasal mucosa, avoiding possible ambiguities. In this work, a web-based app is provided to facilitate the description of the surgery using the LOEM system, which may guide future internal and external validation. Hopefully, this approach will allow reliable information to be easily collected and correlated with comorbidities, prognosis factors, biomarkers, and clinical outcomes in specific CRS cases. The proposed classification system has the potential to translate emerging knowledge into personalized surgical treatments to improve patient outcomes [21].

### Results of an expert pilot study

Using a systematic Delphi method for information prediction over two rounds to reach consensus among experts, the evaluation of our classification proposal resulted in substantial interrater agreement (overall κ = 0.77) (Supplementary Table S3). This iterative process allows for structured feedback, facilitating the refinement of the LOEM classification system based on expert insights and agreement. However, agreement during the first round for the mucosal M subcategory was only fair (κ = 0.37). After a careful revision of the comments provided by the experts, we concluded that the fair agreement reached in this category was due to heterogeneous interpretations of reboot-type surgery among experts [27, 41]. A report based on surgical details of the reboot surgery discussed in the original publication by Alsharif et al. about the difficulty of achieving a complete excision of the mucosa in distal regions of the paranasal sinuses (e.g. the frontal and sphenoid sinuses, or the alveolar recess of the maxillary sinus), due to limitations of visualization and instrumentation [13] was provided and discussed with the experts. Such clarifications allowed us to ensure alignment of the definition of M with the original description of the reboot surgery, which improved the agreement for this section to a value of κ = 0.79 (Supplementary Table S3).

Conversely, decreases in the degree of agreement for O and E were recorded in the second round of the pilot study (κ = 0.52 and κ = 0.41, respectively). This may be due to a misinterpretation between the enlargement of the ostium (O) and the resection of the paranasal sinus wall (E). It is important to note that, in conventional ESS techniques, modifying the extension of the surgery requires a prior adjustment of the sinus ostium. This fact can be unclear when dealing with R-ESS, where previous modifications of the sinus ostia have already been made [42]. Another possible explanation could be the intrinsic limitation of the LOEM classification in adequately defining how much the ostium must be enlarged to consider that an approach to the sinus drainage ostium changes from O to E. To better explain the extension over the different paranasal sinuses, various specific classifications have been used to better define how much the access pathway to each paranasal sinus should be modified (Table 1) [22, 23, 35, 36].

In addition, the intra-rater reliability after 6 months demonstrated a very high reproducibility of the LOEM classification. Our seven experts achieved an overall agreement of 93% and a Kappa index of 0.82, suggesting highly consistent evaluations over time. While exploring the item-specific findings for LOEM, we still found high agreement in all groups, ranging from 82.55% (E items) to 100% (L items). However, item O had an agreement of 91.27%, but the Kappa value was − 0.05 (p = 0.695), indicating no reliability beyond what would be expected by chance. On one hand, this could be due to the calculation's characteristics, where the expected agreement by chance was very high (93%). This high probability of chance agreement can be clinically explained by the strong tendency to perform antrostomies during ESS, directing approaches not only to the location of the drainage ostia but also to their modification, which makes it a practice carried out in nearly all surgical approaches. Furthermore, the fact that the videos selected for the expert pilot study were mostly from expanded surgeries, with the opening of all ostia, may have also overestimated the expected level of agreement. This lack of agreement for the O item suggests that modifications to the LOEM system may be needed to account for challenges in consistent ostium classification. Future large-scale validations planned for the LOEM system should address this discrepancy through further refinement of the system.

It is noteworthy that the internal validation process was strengthened by the knowledge of seven highly experienced rhinologists. They evaluated the surgeries without knowing details about the patients or the operating surgeons, which ensured fair assessments focused solely on surgical outcomes. Additionally, detailed descriptions and sharing of reports after each evaluation round improved transparency and cleared up any uncertainties. Consequently, the LOEM classification system may enable a more precise delineation of different surgical approaches, including more extended mucosal resections. It may also facilitate the inclusion of new regenerative surgery concepts. The implementation and expansion of this system will hopefully enhance consistency in describing surgical approaches for CRS, thereby simplifying comparisons and increasing the reproducibility of the various available techniques.

### Pathway to implementation: integrating the LOEM system into clinical practice

To integrate LOEM into clinical practice and research, the following roadmap can be proposed by authors: (i) develop training programs for surgeons and medical professionals on LOEM, including workshops and online resources to ensure widespread understanding and acceptance; (ii) conduct pilot studies to evaluate the effectiveness of LOEM in various clinical settings, gathering data on its utility and outcomes in patient management; (iii) collaborate with professional organizations to create standardized protocols for the implementation of LOEM in clinical practice; (iv) establish a feedback mechanism to collect user experiences and suggestions for improvement from clinicians using LOEM in practice.

During this process, some issues may arise, such as resistance to change from teams that are accustomed to a specific way of working. There may also be a lack of initial knowledge regarding the interpretation of the various subscripts. Furthermore, a gradual outreach process will be necessary to ensure that future users understand how to use it correctly.

### Limitations, utility and future perspectives

The major limitation of the LOEM system is that it does not consider conservative modalities, such as balloon sinuplasty or technical variations, especially those performed on the inferior and superior turbinates, or nasal septum. Furthermore, it does not consider the treatment of sequelae such as synechiae, mucoceles or osteitis. These are inherent limitations of any classification system, which cannot encompass all aspects and variations of ESS. Nonetheless, the LOEM system remains an easy-to-use, intuitive and valuable tool for describing numerous types of ESS variants in the treatment of CRS, providing a global view of the surgical approach both regarding bony structures and mucosal treatment.

Other limitations inherent to the pilot study include the small sample size of eleven videos, the pilot study conducted in a limited geographical region (Europe), and the exclusion of specific items (L_xa_, O_xa_, E_xa_, O_0_, E_0_) due to case selection criteria, which could restrict the generalizability of findings and limit the broader applicability of the LOEM system globally. Furthermore, potential differences in interpretation and a learning curve among less experienced rhinologists were not fully addressed. This calls for the development of new large-scale validation studies that encompass both seasoned and novice rhinologists, a higher volume of cases, subgroup analyses and additional videos of revision surgeries, to achieve greater robustness and reproducibility of the items included in the LOEM system.

The usefulness of the LOEM classification system lies in the possibility of describing, in a unified way, the different types of ESS performed in the treatment of CRS patients, as well as to compare the different surgical approaches described in the literature. In addition, the LOEM system would help solve current limitations in the use of administrative data based on diagnosis-related groups (DRGs) and International Classification of Diseases (ICDs), due to divergent classification terms used across different organizations and countries [43]. The increasing knowledge on the underlying CRS inflammatory mechanisms and biomarkers, and the solid description of phenotypes in a new precision medicine paradigm, require a systematic classification for ESS extension [17, 44]. In addition, new AI and machine learning algorithms have been developed for predictive modeling of CRS outcomes [45, 46]. The labeling of surgical approaches based on classification systems such as the LOEM system here proposed will promote data acquisition and automated training of AI models in Rhinology.

## Conclusions

The Lamella Ostium Extent Mucosa system suggests a simple but descriptive classification that includes not only surgical extent but also mucosal management. The LOEM system allows different surgical techniques described so far to be integrated under a unified and compact definition. Hopefully, this new classification system will open the door to an effective comparison of surgical outcomes, allowing for a better definition of the appropriate surgical extent for each CRS phenotype.

A pilot study involving expert rhinologists demonstrated an almost perfect agreement when using the LOEM system to describe different surgical CRS cases, underscoring its potential for standardization in clinical practice. Therefore, routine use of LOEM in the workplace may facilitate more accurate descriptions of ESS, improve communication between surgeons, promote more accurate teaching for surgeons-in-training and direct CRS treatment toward personalized medicine.

Additionally, LOEM contributes significantly to the advancement of rhinologic research by providing a standardized language that encourages collaborative studies on surgical techniques and patient management. To maximize its potential, ongoing research and verification of the LOEM system in CRS surgical approaches are essential, promoting collaboration among researchers and clinicians to improve patient outcomes.

## Supplementary Information

Below is the link to the electronic supplementary material.

Supplementary file1 (DOCX 43 kb)

## Data Availability

Not applicable in this section. It is the website included in the manuscript.
