# Supplementary material for: The Lamella Ostium Extent Mucosa (LOEM) system: a new classification and pilot study for endoscopic sinus surgery
Source: Eur Arch Otorhinolaryngol. 2024 Dec 2;282(2):851–67. doi: 10.1007/s00405-024-09092-z (PMC11805853; doi:10.1007/s00405-024-09092-z)
Supplement: Supplementary file 1 — Supplementary file1 (DOCX 43 kb) [file 405_2024_9092_MOESM1_ESM.docx]

| **Supplementary Table S1. The individual expert rhinologists’ responses for each round and rater.** | | | | | | | | | | | | | | | | |
| --- | --- | --- | --- | --- | --- | --- | --- | --- | --- | --- | --- | --- | --- | --- | --- | --- |
| **ROUND 1** | | | | | | | | | | | | | | | | |
|  |  | **L_0_** | **L_1_** | **L_2_** | **L_3_** | **O_m_** | **O_f_** | **O_s_** | **E_m_** | **E_f_** | **E_s_** | **E_t_** | **M_0_** | **M_f_** | **M_r_** | **M_m_** |
| **CASE 1** | **Expert 1** | - | + | + | + | + | + | + | + | - | + | + | - | + | - | - |
|  | **Expert 2** | - | + | + | + | + | + | + | + | - | + | + | - | - | + | - |
|  | **Expert 3** | NR | NR | NR | NR | NR | NR | NR | NR | NR | NR | NR | NR | NR | NR | NR |
|  | **Expert 4** | - | + | + | + | + | + | + | + | - | + | + | - | + | - | - |
|  | **Expert 5** | - | + | + | + | + | + | + | + | - | + | + | - | + | - | - |
|  | **Expert 6** | - | + | + | + | + | + | + | + | - | + | + | - | + | - | - |
|  | **Expert 7** | - | + | + | + | + | + | + | + | - | + | + | - | + | - | - |
| **CASE 2** | **Expert 1** | - | + | + | + | + | + | + | + | - | + | + | - | + | - | - |
|  | **Expert 2** | - | + | + | + | + | + | + | + | - | + | + | - | - | + | - |
|  | **Expert 3** | NR | NR | NR | NR | NR | NR | NR | NR | NR | NR | NR | NR | NR | NR | NR |
|  | **Expert 4** | - | + | + | + | + | + | + | + | - | + | + | - | + | - | - |
|  | **Expert 5** | - | + | + | + | + | + | + | + | - | + | + | - | + | - | - |
|  | **Expert 6** | - | + | + | + | + | + | + | + | - | + | + | - | + | - | - |
|  | **Expert 7** | - | + | + | + | + | + | + | + | - | + | + | - | + | - | - |
| **CASE 3** | **Expert 1** | - | + | + | + | + | + | + | - | + | - | + | - | - | + | - |
|  | **Expert 2** | - | + | + | + | + | + | + | + | - | + | + | - | - | + | - |
|  | **Expert 3** | NR | NR | NR | NR | NR | NR | NR | NR | NR | NR | NR | NR | NR | NR | NR |
|  | **Expert 4** | - | + | + | + | + | + | + | - | - | - | + | - | + | - | - |
|  | **Expert 5** | - | + | + | + | + | + | - | + | + | - | + | - | - | + | - |
|  | **Expert 6** | - | + | + | + | + | + | - | + | + | - | + | - | - | + | - |
|  | **Expert 7** | - | + | + | + | + | + | - | + | - | - | + | - | + | - | - |
| **CASE 4** | **Expert 1** | - | + | + | + | + | + | + | + | - | + | + | - | - | + | - |
|  | **Expert 2** | - | + | + | + | + | + | + | + | - | + | + | - | - | + | - |
|  | **Expert 3** | - | + | + | + | + | + | + | + | - | + | + | - | - | - | + |
|  | **Expert 4** | - | + | + | + | + | + | + | + | - | + | + | - | - | + | - |
|  | **Expert 5** | - | + | + | + | + | + | + | + | + | + | + | - | - | - | + |
|  | **Expert 6** | - | + | + | + | + | + | + | + | - | + | + | - | - | - | + |
|  | **Expert 7** | - | + | + | + | + | + | + | + | + | + | + | - | - | - | + |
| **CASE 5** | **Expert 1** | - | + | + | + | + | + | + | + | - | + | + | - | - | + | - |
|  | **Expert 2** | - | + | + | + | + | + | + | + | - | + | + | - | - | + | - |
|  | **Expert 3** | - | + | + | + | + | + | + | - | - | + | + | - | - | - | + |
|  | **Expert 4** | - | + | + | + | + | + | + | + | - | + | + | - | - | + | - |
|  | **Expert 5** | - | + | + | + | + | + | + | + | - | + | + | - | - | - | + |
|  | **Expert 6** | - | + | + | + | + | + | + | + | - | + | + | - | - | - | + |
|  | **Expert 7** | - | + | + | + | + | + | + | + | + | + | + | - | - | - | + |
| **ROUND 2** | | | | | | | | | | | | | | | | |
|  |  | **L_0_** | **L_1_** | **L_2_** | **L_3_** | **O_m_** | **O_f_** | **O_s_** | **E_m_** | **E_f_** | **E_s_** | **E_t_** | **M_0_** | **M_f_** | **M_r_** | **M_m_** |
| **CASE 6** | **Expert 1** | - | + | + | + | + | + | + | + | + | + | + | - | - | - | + |
|  | **Expert 2** | - | + | + | + | + | + | + | + | + | + | - | - | - | - | + |
|  | **Expert 3** | - | + | + | + | + | + | + | + | - | + | - | - | - | - | + |
|  | **Expert 4** | - | + | + | + | + | + | + | + | - | + | + | - | - | - | + |
|  | **Expert 5** | - | + | + | + | + | + | + | + | - | + | + | - | - | - | + |
|  | **Expert 6** | - | + | + | + | + | + | + | + | - | + | + | - | - | - | + |
|  | **Expert 7** | - | + | + | + | - | + | - | + | - | + | - | - | - | - | + |
| **CASE 7** | **Expert 1** | - | + | + | + | + | + | + | + | + | + | + | - | - | - | + |
|  | **Expert 2** | - | + | + | + | + | + | + | + | + | + | - | - | - | - | + |
|  | **Expert 3** | - | + | + | + | + | + | + | + | - | + | - | - | - | - | + |
|  | **Expert 4** | - | + | + | + | + | + | + | + | - | + | + | - | - | - | + |
|  | **Expert 5** | - | + | + | + | + | + | + | + | - | + | + | - | - | - | + |
|  | **Expert 6** | - | + | + | + | + | + | + | + | - | + | + | - | - | - | + |
|  | **Expert 7** | - | + | + | + | - | + | - | + | - | + | - | - | - | - | + |
| **CASE 8** | **Expert 1** | - | + | + | + | + | + | + | + | + | + | + | - | + | - | - |
|  | **Expert 2** | - | + | + | + | + | + | + | + | + | + | + | - | + | - | - |
|  | **Expert 3** | - | + | + | + | + | + | + | + | + | + | + | - | + | - | - |
|  | **Expert 4** | - | + | + | + | + | + | + | + | - | + | + | - | + | - | - |
|  | **Expert 5** | - | + | + | + | + | + | + | + | - | + | + | - | + | - | - |
|  | **Expert 6** | - | + | + | + | + | + | + | + | - | - | + | - | + | - | - |
|  | **Expert 7** | - | + | + | + | + | + | + | + | + | + | + | - | + | - | - |
| **CASE 9** | **Expert 1** | - | + | + | + | + | + | + | + | + | + | + | - | + | - | - |
|  | **Expert 2** | - | + | + | + | + | + | + | + | + | + | + | - | + | - | - |
|  | **Expert 3** | - | + | + | + | + | + | + | + | + | + | + | - | + | - | - |
|  | **Expert 4** | - | + | + | + | + | + | + | + | - | + | + | - | + | - | - |
|  | **Expert 5** | - | + | + | + | + | + | + | + | - | + | + | - | + | - | - |
|  | **Expert 6** | - | + | + | + | + | - | + | + | - | - | + | - | + | - | - |
|  | **Expert 7** | - | + | + | + | + | + | + | + | + | + | + | - | + | - | - |
| **CASE 10** | **Expert 1** | - | + | + | + | + | + | + | + | + | + | + | - | - | + | - |
|  | **Expert 2** | - | + | + | + | + | + | + | + | - | + | + | - | + | - | - |
|  | **Expert 3** | - | + | + | + | + | + | + | + | - | - | + | - | + | - | - |
|  | **Expert 4** | - | + | + | + | + | + | + | + | - | + | + | - | + | - | - |
|  | **Expert 5** | - | + | + | + | + | + | + | + | - | + | + | - | + | - | - |
|  | **Expert 6** | - | + | + | + | + | + | + | + | - | + | + | - | + | - | - |
|  | **Expert 7** | - | + | + | + | + | + | + | + | - | + | + | - | - | + | - |
| **CASE 11** | **Expert 1** | - | + | + | + | + | + | + | + | + | + | + | - | - | + | - |
|  | **Expert 2** | - | + | + | + | + | + | + | + | - | + | + | - | + | - | - |
|  | **Expert 3** | - | + | + | + | + | + | + | + | - | - | + | - | + | - | - |
|  | **Expert 4** | - | + | + | + | + | + | + | + | - | + | + | - | + | - | - |
|  | **Expert 5** | - | + | + | + | + | + | + | + | - | + | + | - | + | - | - |
|  | **Expert 6** | - | + | + | + | + | + | + | + | - | + | + | - | + | - | - |
|  | **Expert 7** | - | + | + | + | + | + | + | + | - | + | + | - | - | + | - |
| L0: No intervention is performed on the anterior and posterior ethmoid structures. L1: The first lamella involves the removal of the uncinate process and the agger nasi cell, along with other anterior ethmoidal cells that are superior to the agger nasi if present. L2: The second lamella refers to the removal of the ethmoid bulla, as well as any suprabullar and retrobullar cells and recesses that may be present. L3: The third lamella includes the opening of the vertical plate of the basal lamella of the middle turbinate and removing any bony septa of the posterior ethmoidal cells. O_0_: No intervention is performed on any of the sinus ostia / opening. Om: Indicates enlargement of the maxillary sinus ostium. Of: Refers to techniques used in the Draf I and IIa frontal sinus surgery or Grades 0 to 4 of the Classification of the Extent of Endoscopic Frontal Sinus Surgery (EFSS). Os: Includes enlargement techniques performed on the sphenoid sinus ostium, regardless of the specific surgical approach used. E0: No extended intervention is performed on the walls of any large sinus. Em: Significant widening of the medial wall of the maxillary sinus. Ef: Considerable opening of the frontal sinus floor, which can be achieved through Draf IIb or Draf III techniques or graded as 5 or 6 according to the Classification of the Extent of Endoscopic Frontal Sinus Surgery (EFSS). Es: Substantial enlargement of the anterior wall of the sphenoid sinus. Et: Partial removal or trimming of the middle turbinate. M0: Cases with no mucosal removal, except for the simple enlargement of the sinus ostia / openings. Mf: If the main goal is to preserve mucociliary function, regardless of the extent of bone surgery performed. This category encompasses the original criterion of FESS that aimed to remove only macroscopically mainly irreversibly diseased mucosa. Mr: If the goal is to replace all sinus mucosa with healthy locally mucosa, regardless of the extent of bone surgery performed. Mm: If the intention is to create neomucosa from freely grafted healthy mucosa covering a significant part of the excised mucosal area in addition to radical ESS.  *Abbreviations*: (**+**) Yes; (**-**) No; **NR** = No response. | | | | | | | | | | | | | | | | |

| **Supplementary Table S2. Qualitative comments made by experts on LOEM's ease of use and completeness.** | |
| --- | --- |
| **Expert 1** | The LOEM classification has a clear structure, which facilitates its understanding and application in clinical practice. The generated web app is easy to use and aids in extending the classification; however, I believe it would be helpful to provide more detailed training materials to help surgeons quickly familiarize themselves with the new criteria. |
| **Expert 2** | The classification covers a wide range of clinical presentations. The concepts of lamella, ostium, extension, and mucosa address the fundamentals of ESS. I would consider whether the LOEM classification could benefit from greater comprehensiveness in certain complex cases that may not currently be covered. |
| **Expert 3** | The LOEM successfully encompasses the principles of endoscopic surgery. I believe this new system can facilitate communication among healthcare professionals, which is essential in the multidisciplinary context of chronic rhinosinusitis. However, I wonder how easily this classification integrates into the usual workflow of surgeons. |
| **Expert 4** | The classification appears adaptable to various surgical scenarios, which is an advantage. Compared to existing classifications, I believe LOEM addresses some gaps, but more clarity is needed in certain aspects related to mucosal treatment. |
| **Expert 5** | The logic of the LOEM structure is evident and can facilitate its use, but some surgeons may require an adjustment period. It would be useful to conduct a more thorough review and consider feedback from non-European rhinologists in future versions. |
| **Expert 6** | The LOEM provides clear definitions that are crucial for differentiating various surgical extensions, thereby improving decision-making during surgery. However, I am concerned that some surgeons may encounter obstacles in applying the classification despite the web app. It would also be interesting to explore how artificial intelligence could complement this classification in the future. |
| **Expert 7** | The classification is relevant and provides a useful framework for clinical decision-making. I believe it is important to conduct ongoing evaluation of the classification in the future to ensure it remains updated with the latest evidence. I am convinced that, over time, LOEM can become an essential tool for endoscopic surgery. |

| **Supplementary Table S3. LOEM agreement between rhinologists** | | | |
| --- | --- | --- | --- |
|  | **Kappa** | **Z** | ***p* value*** |
| **Round 1** | | | |
| **Whole items** | 0.77 (0.72-0.85) | 27.67 | <0.0001 |
| **L** | 1.00 (NA) | 18.59 | <0.0001 |
| **O** | 0.66 (0.34-0.82) | 10.57 | <0.0001 |
| **E** | 0.68 (0.53-0.78) | 12.58 | <0.0001 |
| **M** | 0.37 (0.29-0.45) | 6.79 | <0.0001 |
| **Round 2** | | | |
| **Whole items** | 0.81 (0.78-0.83) | 35.07 | <0.0001 |
| **L** | 1.00 (NA) | 18.59 | <0.0001 |
| **O** | 0.52 (0.44-0.60) | 10.14 | <0.0001 |
| **E** | 0.41 (0.24-0.48) | 9.22 | <0.0001 |
| **M** | 0.79 (0.69-0.82) | 17.70 | <0.0001 |
| **p* value was determined through Cohen’s Kappa. NA (not applicable). Between brackets, 95% confidence interval. | | | |

| **Supplementary Table S4. Test-retest analysis between rounds for the same rhinologists** | | | | | |
| --- | --- | --- | --- | --- | --- |
|  | **Agreement** | **Expected agreement** | **Kappa** | ***p* value*** | |
| **Whole items** | 92.96% | 54.72% | 0.82 | <0.001 | |
| **L** | 100% | 62.50% | 1.00 | <0.001 | |
| **O** | 91.27% | 92.65% | -0.05 | 0.695 | |
| **E** | 81.55% | 60.76% | 0.53 | <0.001 | |
| **M** | 95.24% | 62.50% | 0.87 | <0.001 | |
| **p* value was determined through Cohen’s Kappa | | | | |  |
